# Supplementary material for: The Effect of Lower Limb Combined Neuromuscular Electrical Stimulation on Skeletal Muscle Signaling for Glucose Utilization, Myofiber Distribution, and Metabolic Function after Spinal Cord Injury
Source: Int J Environ Res Public Health. 2023 Oct 21;20(20):6958. doi: 10.3390/ijerph20206958 (PMC10606374; doi:10.3390/ijerph20206958)

Supplemental Information: Western Blots

Glucose transporter type 4 (GLUT4) – 48 kD

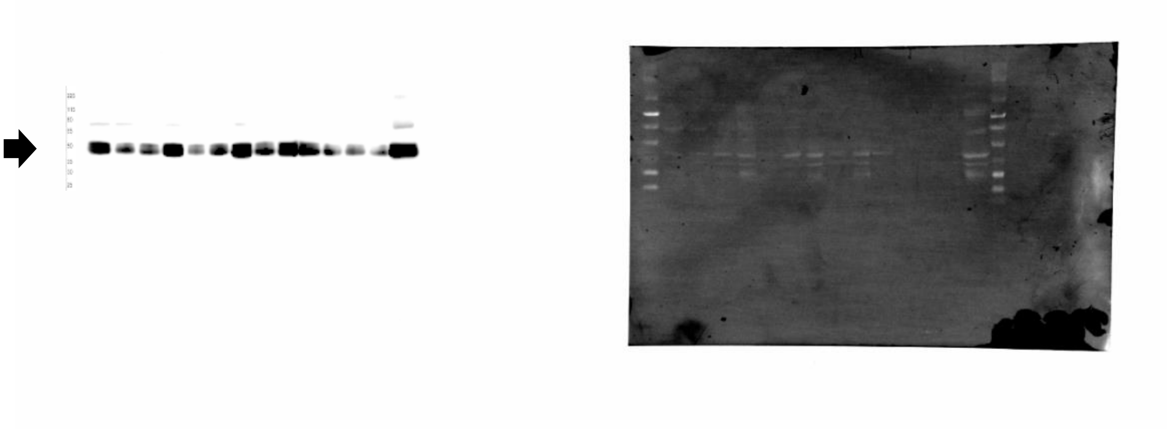

Gel 1 and 2, 4-12% Bis-Tris

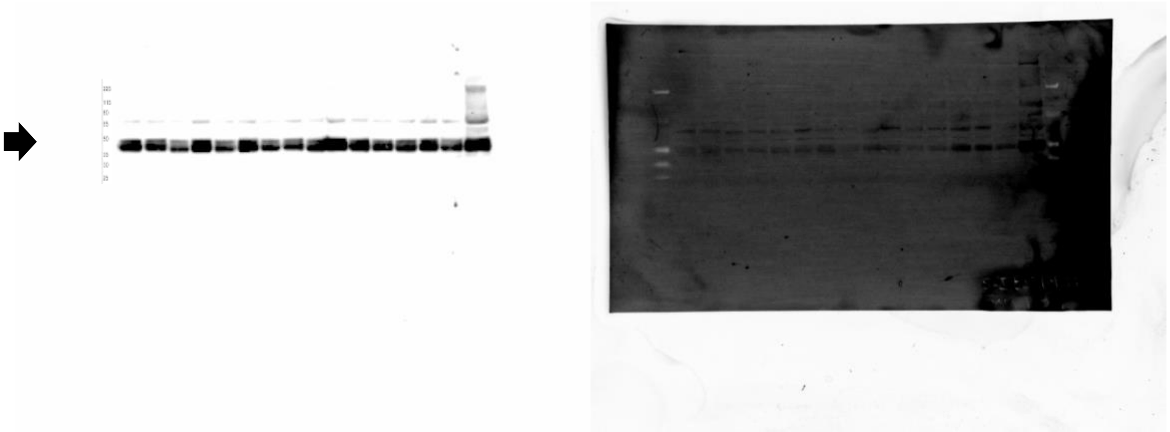

AMP-activated protein kinase, subunit alpha (AMPK-α) – 62 kD

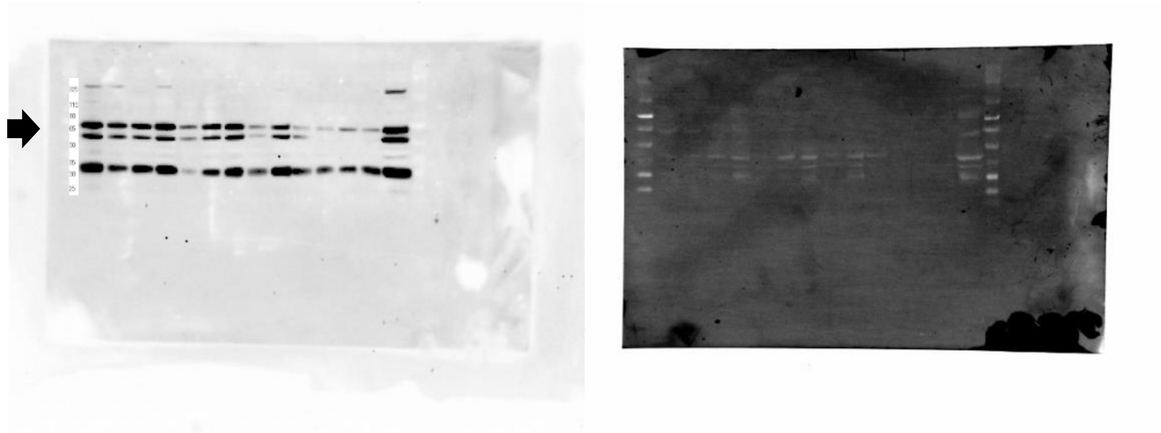

Gel 1 and 2, 4-12% Bis-Tris

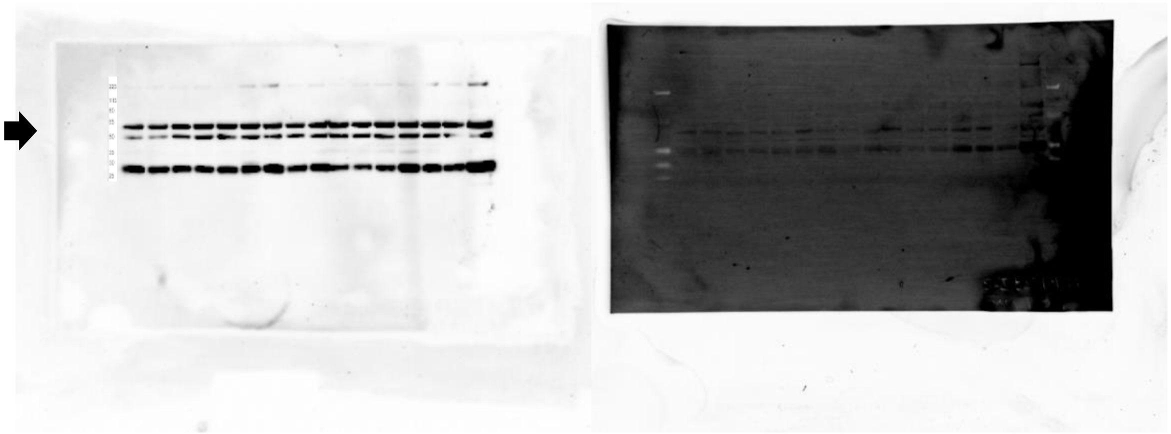

phosphorylated AMP-activated protein kinase, subunit alpha (Thr172) (p-AMPK-α Thr172) – 62 kD

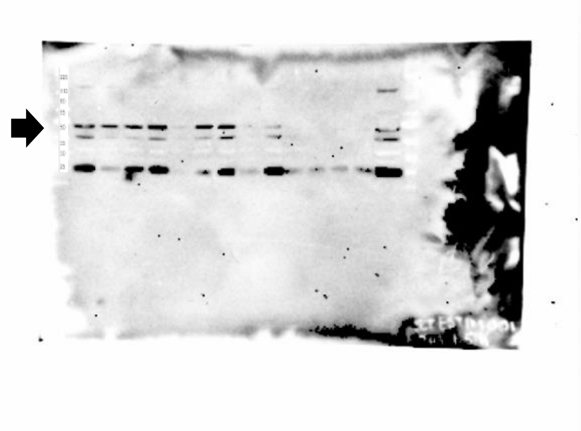
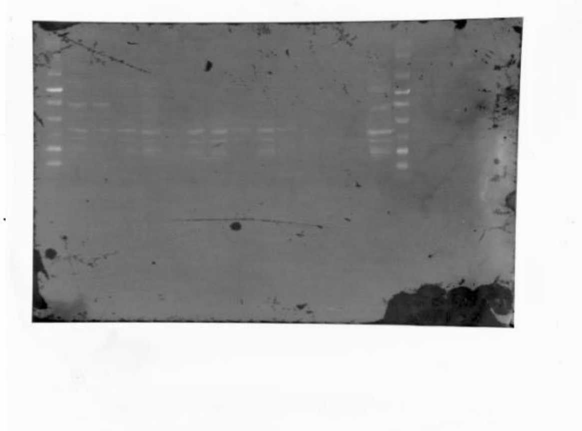

Gel 1 and 2, 4-12% Bis-Tris

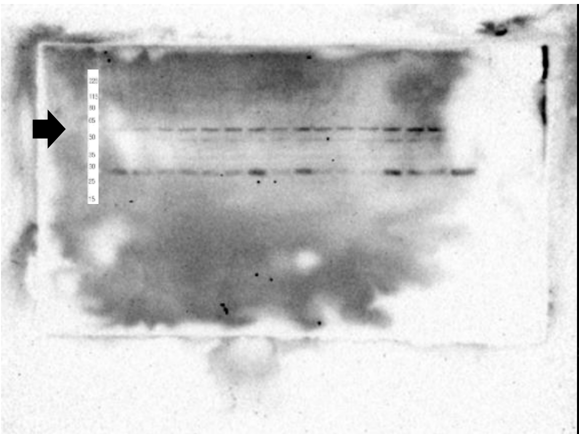
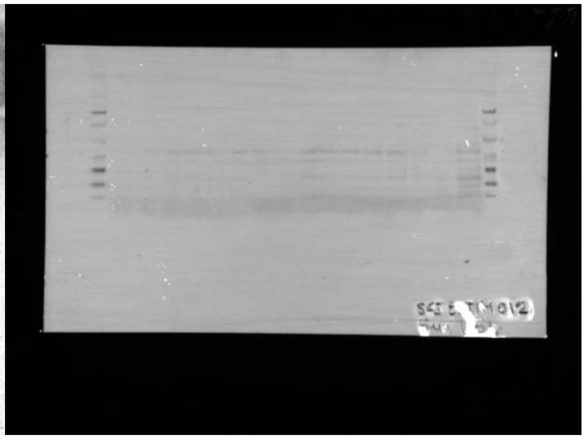

Calcium–calmodulin (CaM)-dependent protein kinase II (CaMKII) – 50 kD

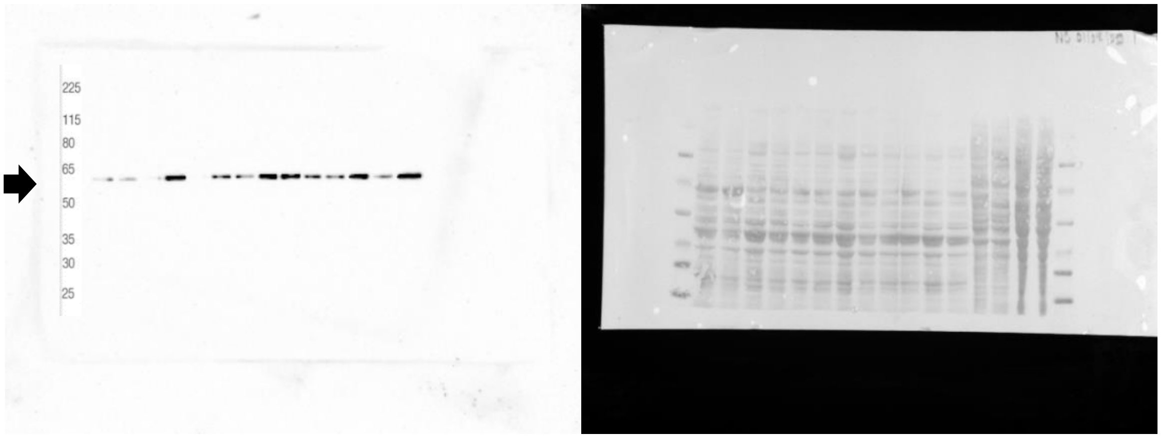

Gel 1 and 2, 4-12% Bis-Tris

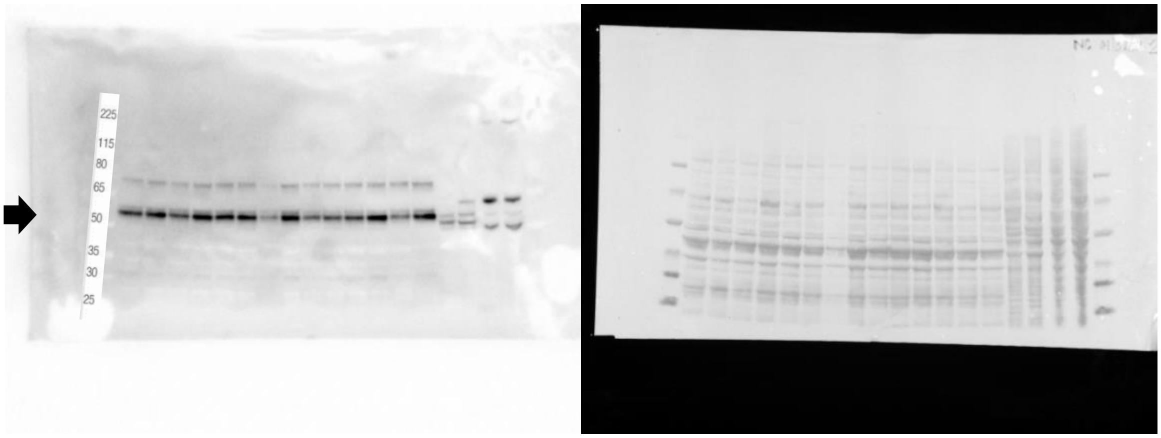

phosphorylated-Calcium–calmodulin (CaM)-dependent protein kinase II Thr286 (p-CaMKII Thr286) – 50 kD

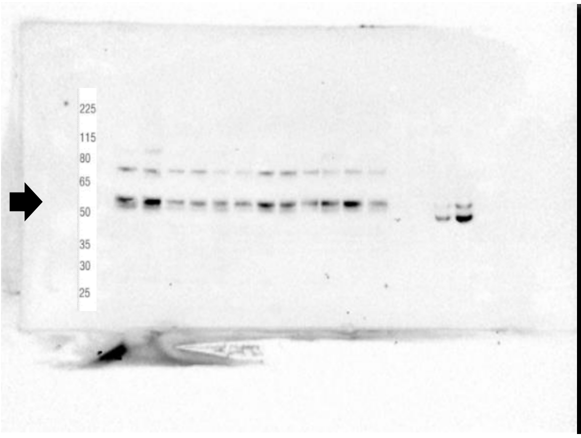

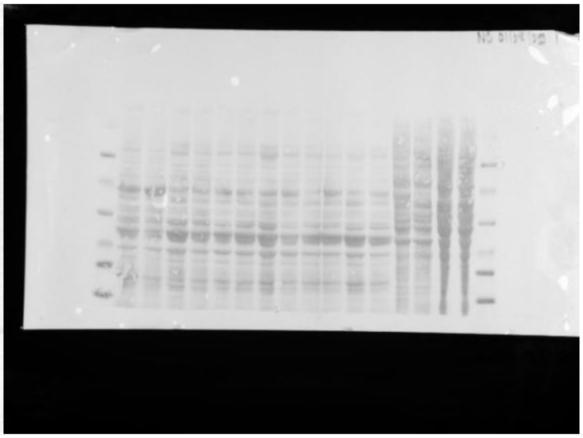

Gel 1 and 2, 4-12% Bis-Tris

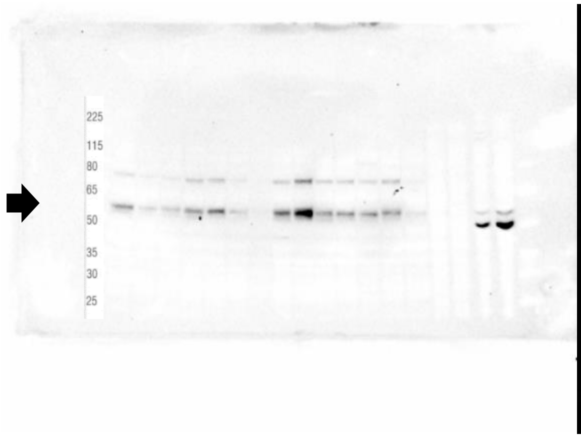

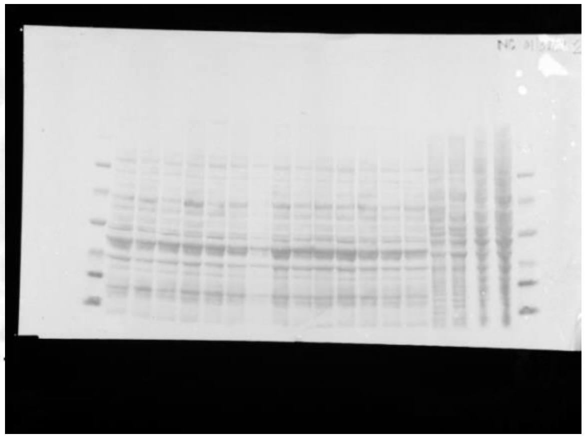

Protein kinase B (AKT) – 60 kD

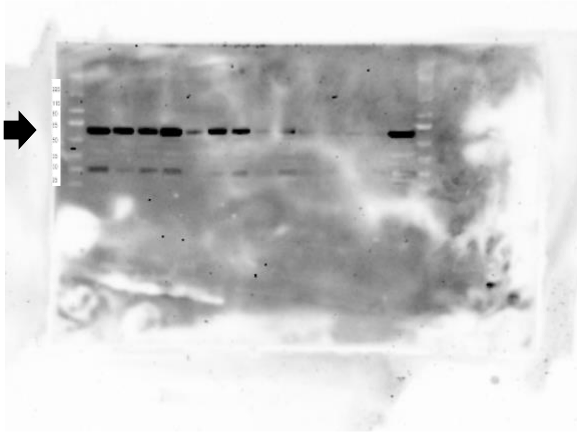
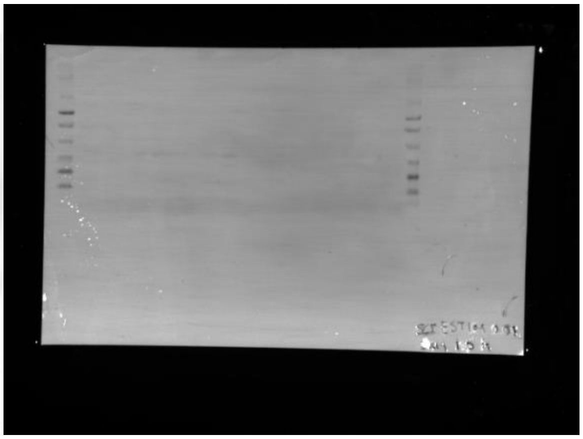

Gel 1 and 2, 4-12% Bis-Tris

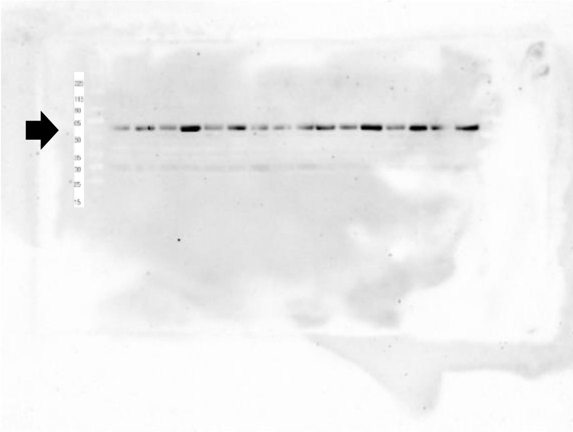
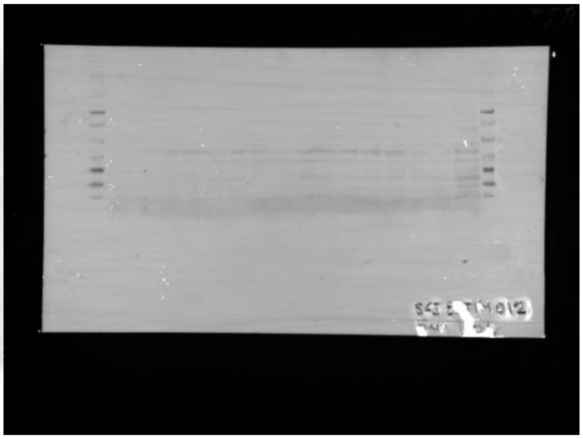

phosphorylated-Protein kinase B (AKT) Ser473 (p-AKT Ser473) – 60 kD

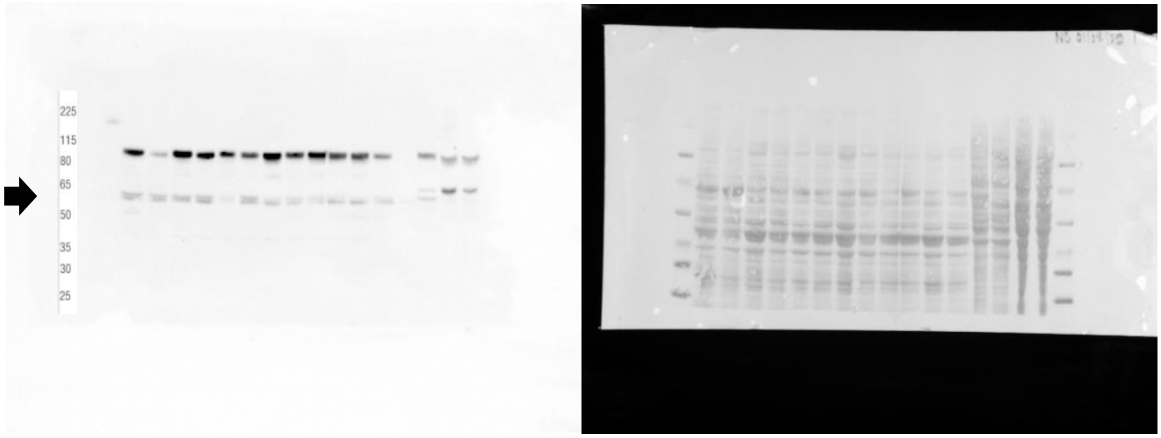

Gel 1 and 2, 4-12% Bis-Tris

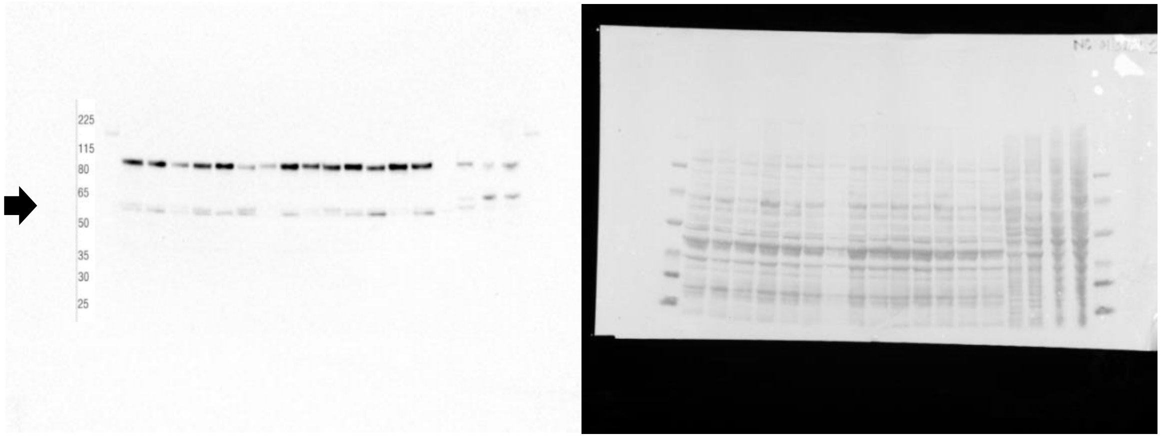

AS160 (AKT substrate of 160 kD)

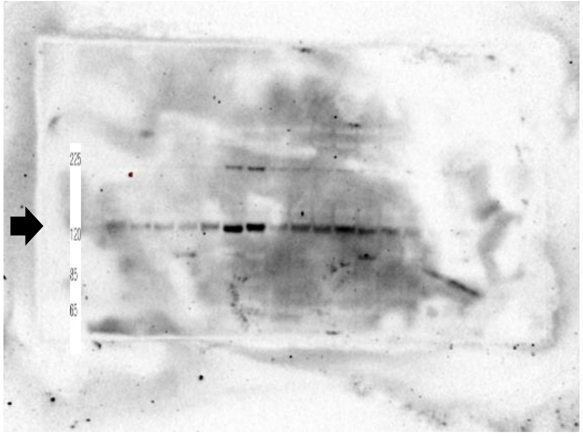

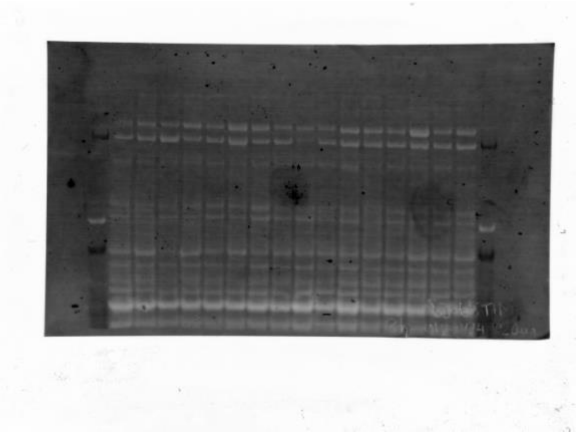

Gel 1 and 2, 3-8% Tris-Acetate

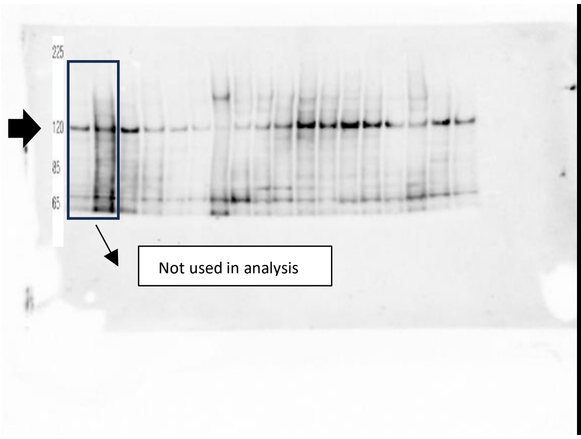

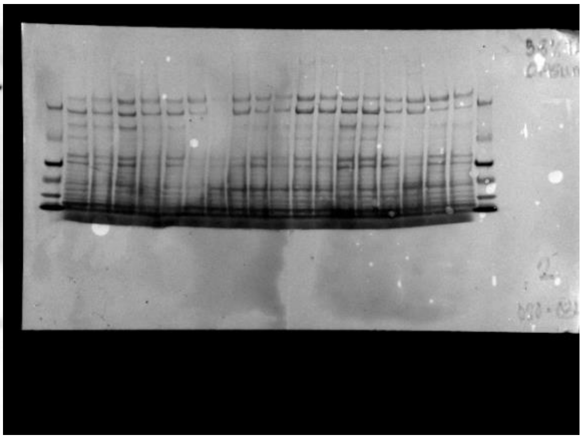

phosphorylated-AS160 Thr642 (p-AS160 Thr642) – 160 kD

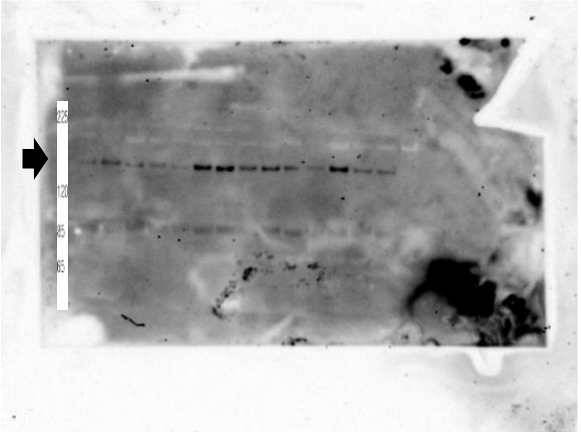

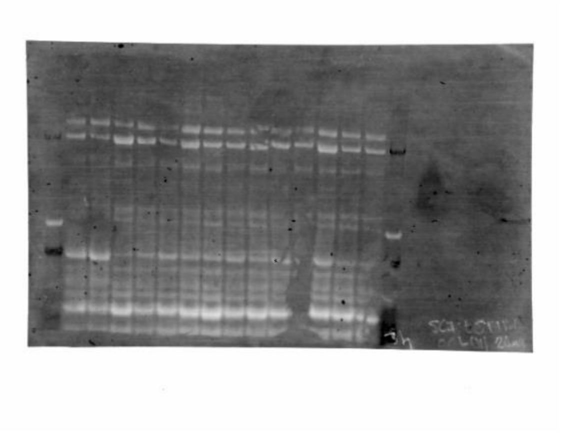

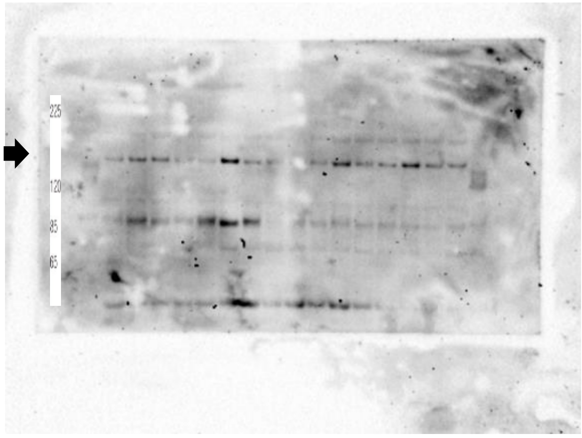

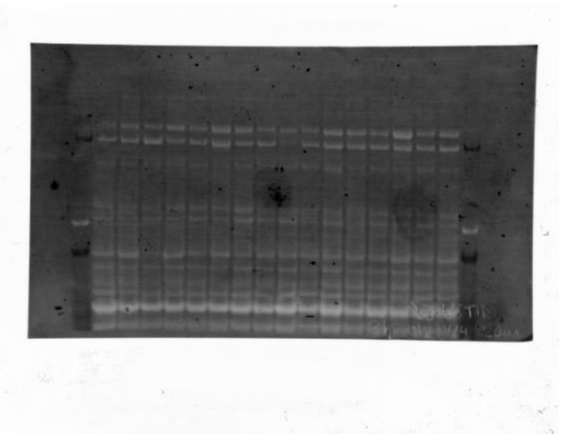

Hexokinase II – 102 kD

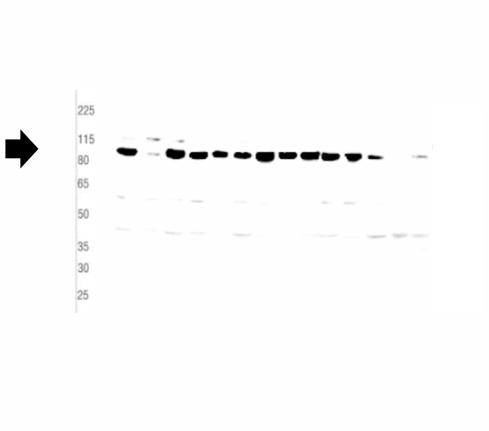

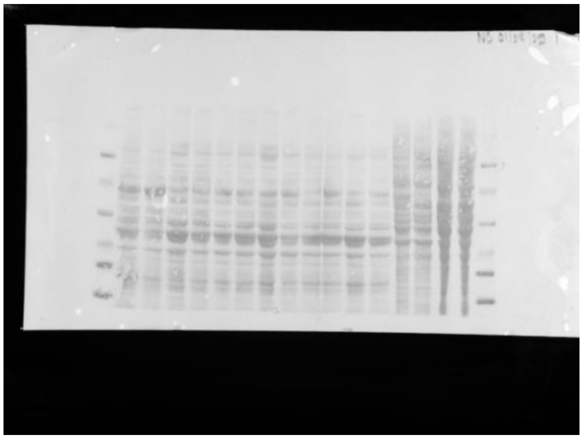

SCI-ESTIM 001-011 plus controls, 4-12% Bis-Tris

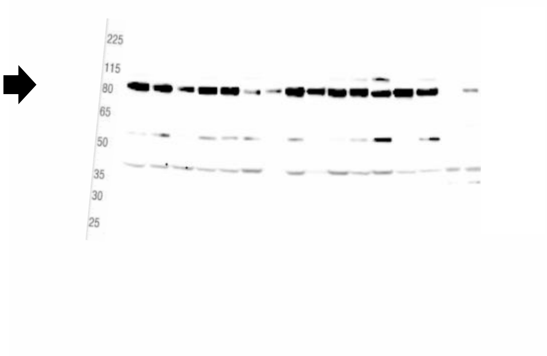

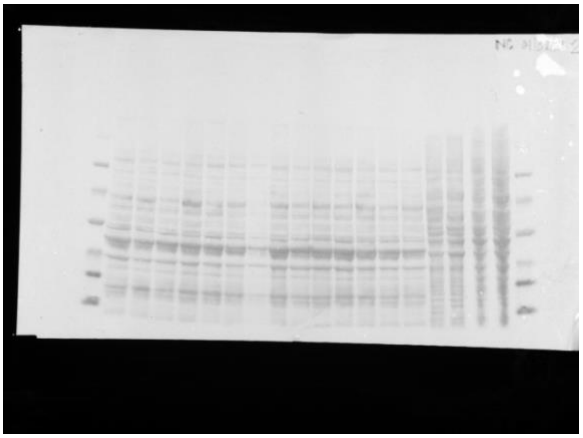

Insulin receptor substrate 1 (IRS-1) – 180 kD

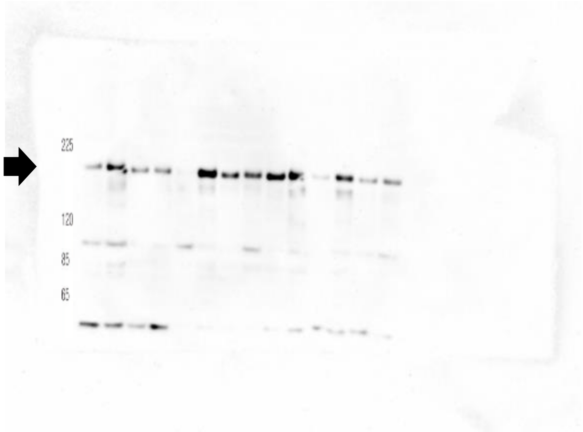

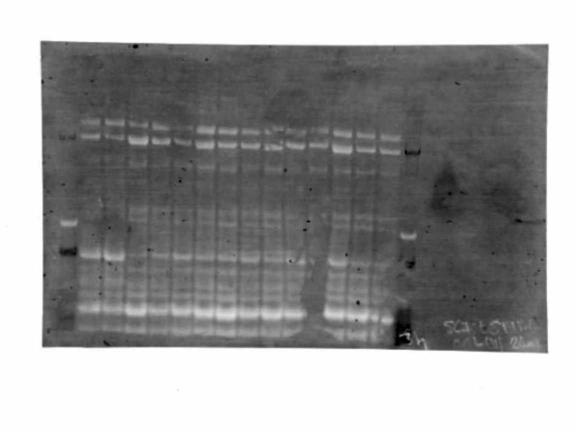

Gel 1 and 2, 3-8% Tris-Acetate

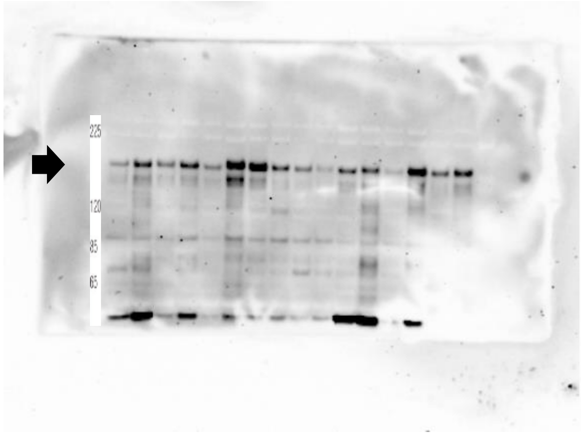

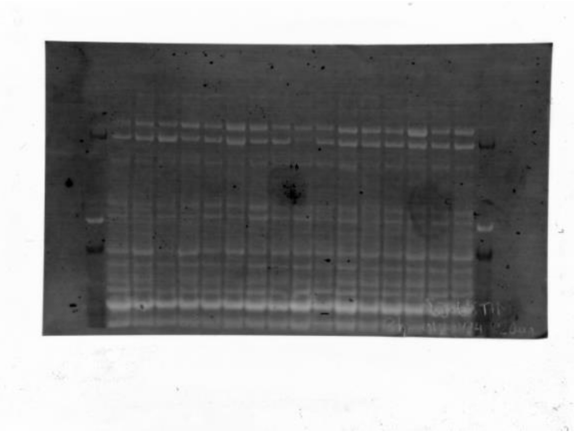

phosphorylated-Insulin receptor substrate 1 (Tyr895) (p-IRS-1 Tyr895) – 180 kD

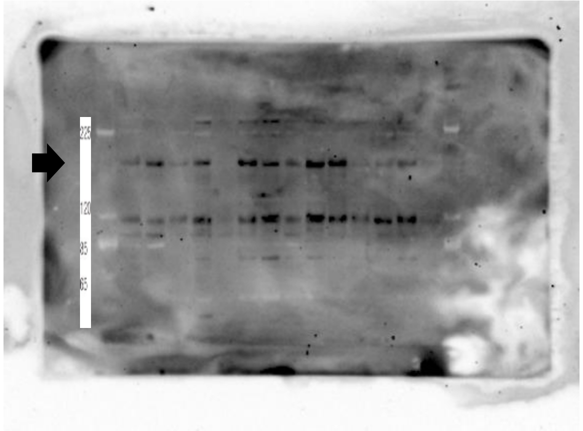

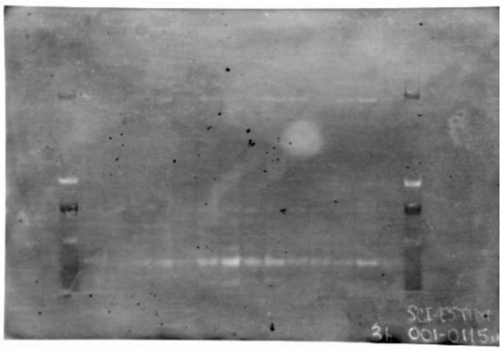

Gel 1 and 2, 3-8% Tris-Acetate

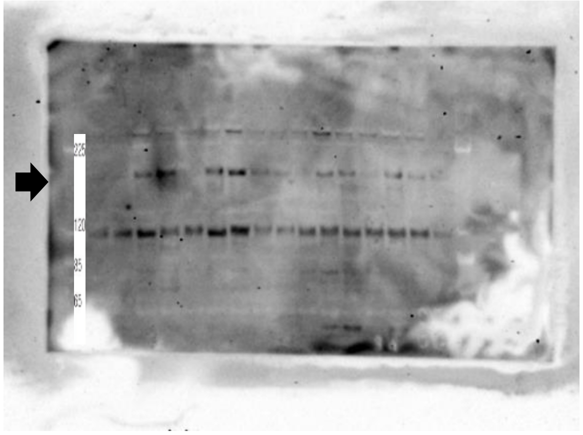

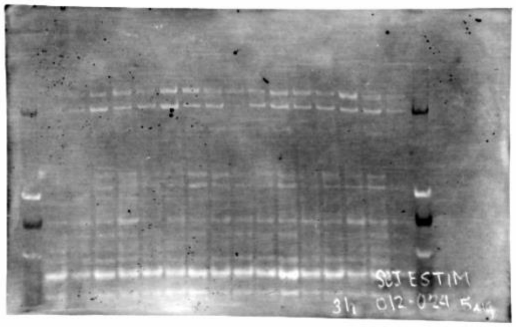

Glycogen Synthase – 84 kD

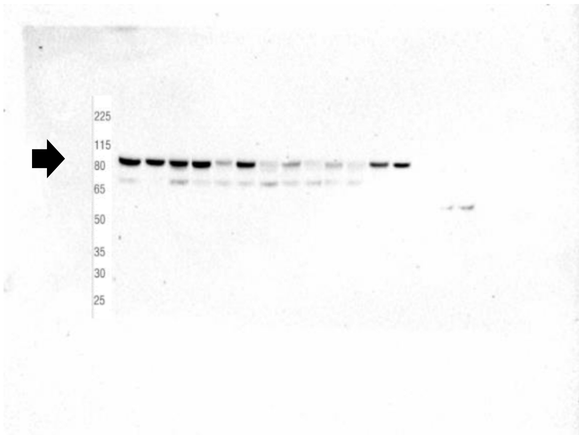

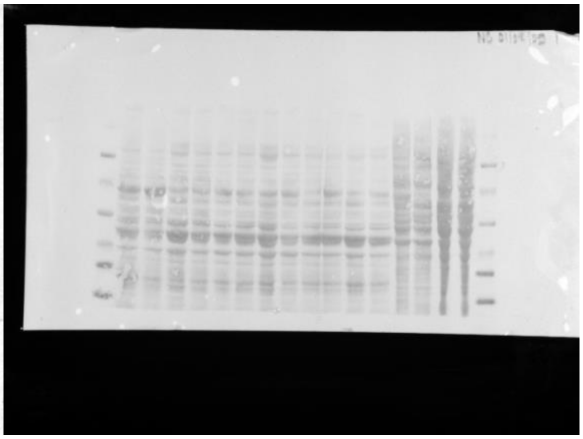

Gel 1 and 2, 4-12% Bis-Tris

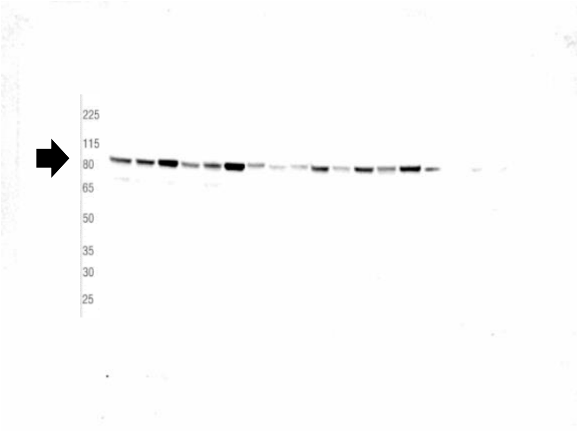

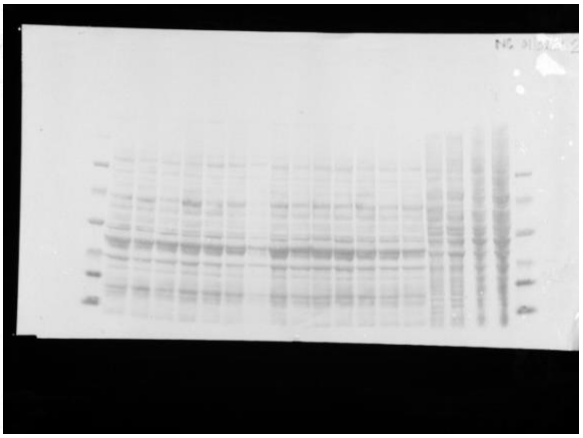

Supplement: Supplementary file 1 [file ijerph-20-06958-s001.zip › ijerph-2606122-supplementary.pdf]
